# Risk and Benefit of Decreasing Seafood Consumption in Japan—Docosahexaenoic Acid, Methylmercury and Infant IQ

**DOI:** 10.3390/foods12081674

**Published:** 2023-04-17

**Authors:** Shingo Fujimura, Jun Yoshinaga

**Affiliations:** Faculty of Biosciences, Toyo University, Izumino 1-1-1, Itakura, Oura, Gunma 374-0193, Japan; volley1232r@gmail.com

**Keywords:** seafood, DHA, methylmercury, woman of childbearing age, infant IQ

## Abstract

National statistics show that seafood consumption in Japan is decreasing since the mid-1990s. The risks and benefits of this decreasing seafood consumption was assessed in this study. Intake of docosahexaenoic acid (DHA) and methylmercury (MeHg) of women of childbearing age were estimated by using seafood consumption data of women of age 20–39 in the period 2011–2019 and seafood DHA and MeHg content data to find significantly (*p* < 0.05) decreasing intake of DHA (2.8 mg/day per year) and MeHg (0.19 μg Hg/day per year) in this period. The effect of the decreasing maternal DHA and MeHg intake on infant IQ was estimated by using the equation developed by the FAO/WHO. Net IQ change (the difference in IQ gain by DHA and IQ loss by MeHg) was constant or even increasing, depending on the assumption, in this period while seafood consumption was significantly decreasing. This was due to the decreasing adverse effect of MeHg, along with saturated DHA-derived benefits on infant IQ, even at the decreased seafood consumption of Japanese women of childbearing age. It was indicated that the recent decreasing trend in seafood consumption in Japan did not have an unfavorable effect on infant IQ.

## 1. Introduction

The National Health and Nutrition Survey (NHNS) of the Ministry of Health, Labor and Welfare of Japan (MHLW) [1] revealed that daily seafood (fish, shellfish and their products) consumption of the Japanese has decreased from the end of the last century to the present. After World War II, the seafood consumption of the Japanese gradually increased to reach the maximum (98.2 g/person/day) in 1997. The consumption subsequently decreased steadily to 63.9 g/person/day in 2019 (Figure S1); an approximately 35% decrease in the last 20 years. The health impact of this decrease is of interest.

The decreasing seafood consumption was paralleled with increasing meat consumption, so the daily protein intake level in the last two decades has been relatively constant at around 70 g/person/day. From the nutritional viewpoint, one of the most important issues potentially related to this decrease in seafood consumption is the associated decrease in the intake of n-3 polyunsaturated fatty acid (PUFA). In fact, there has been a decreasing trend in n-3 PUFA intake from 2005 to the present in Japan. Seafood is a rich source of PUFA, though not the sole source. PUFA is known to be related with decreased incidence of cardiovascular diseases in adults and the enhanced cognitive development of infants [2,3,4,5]. On the other hand, seafood, which includes animals at higher trophic levels in the marine environment, is also known to be a significant source of biomagnifying toxic substances, e.g., methylmercury (MeHg). Seafood is virtually the sole dietary source of MeHg for the general population. It is known that the most sensitive adverse effect caused by MeHg is the developmental delay of infants born to mothers with chronic, excessive intake [6]. A tolerable daily intake of MeHg has been determined based on this adverse effect in many countries.

Thus, the decreasing seafood consumption of the Japanese can have both adverse and beneficial effects on the cognitive development of infants due to decreased intake of PUFA, particularly that of docosahexaenoic acid (DHA), and the decreased intake of MeHg, respectively, of their mother during pregnancy. The aim of this study was to quantitatively estimate adverse and beneficial effects, and the net effect, on the IQ of infants due to decreased seafood consumption of pregnant Japanese women. Average DHA and MeHg intakes from seafood and their temporal trend were estimated by using the national statistics on the intake of the categories of seafood and published DHA and MeHg concentrations of fish and shellfish species and products. The effect of the DHA and MeHg intake of Japanese women of childbearing age on infant IQ was calculated with an equation developed by the FAO/WHO [7], and its temporal change during the period of decreasing seafood consumption was evaluated. To date, the quantitative association between maternal seafood consumption and the Infant Developmental Index score has not been extensively examined in an epidemiologic study carried out in Japan [8]. Estimating the quantitative relationship is relevant to evaluating the past and present situations and predicting future trends of the developmental effect of seafood consumption.

## 2. Materials and Methods

### 2.1. Seafood Categories

Daily consumption of 13 categories of seafood by the Japanese population is available in the results of the NHNS, MHLW, which has been conducted yearly by involving randomly selected persons in Japan (typically 6000–9000 persons). The categories of seafood include 8 seafood categories (“horse mackerel and sardine”, “salmon and trout”, “sea bream and flatfish”, “tuna and swordfish”, “other fish”, “shellfish”, squid and octopus”, and “shrimp and crab”) and 5 seafood products (“salted fish”, “canned fish”, “*tsukudani*” (seafood boiled in sweetened soy sauce), “fish paste” and “fish ham and sausage”) categories. Supplemental Table S1 shows consumption of the 13 seafood categories by women aged 20–29 and aged 30–39, who are tentatively designated as “women of childbearing age” hereafter, in 2019, as an example. Each category includes a variety of items, which are exemplified by the MHLW.

### 2.2. DHA and MeHg Contents of Seafood Categories

The docosahexaenoic acid content of seafood and seafood products was obtained from the Standard Tables of Food Composition (8th Revised Version) [9]. The methylmercury content of seafood and seafood products was obtained from the literature [10,11,12,13,14,15]. In total, 449 sets of MeHg data were collected from the literature; each data set consisted of 1 to 120 individual data. No DHA and MeHg content of food items belonging to the “fish ham and sausage” category was available in the literature; therefore, the mean content of DHA and MeHg of the other four categories of seafood products was substituted for this category. The methylmercury content of seafood has usually been reported for raw fish and shellfish, while the NHNS seafood consumption data has been expressed on a cooked seafood basis. The methylmercury content of cooked seafood was estimated by correcting the moisture content of the raw and cooked items, which were obtained from the Standard Tables of Food Composition [9]. Docosahexaenoic acid contents are listed as the content in cooked seafood in the Standard Table of Food Composition.

### 2.3. Estimated DHA and MeHg Intake

The average DHA and MeHg contents of seafood of the 13 categories were obtained by averaging the DHA and MeHg contents of fish and shellfish species belonging to each of the categories. Daily intakes of DHA (mg/day) and MeHg (μg Hg/day) of women of childbearing age in 2011–2019 were calculated by multiplying the average contents of DHA (mg/g) and MeHg (μg Hg/g) by daily consumption for each of the 13 seafood categories (g/day) (see Table S1 for reference), and by summing them all. Calculation was limited to 2011–2019 because data on sex and age divided by the 13-category seafood consumption are available only from 2011 in the NHNS reports. Seafood consumption of women of age 20–29 and 30–39 were averaged and regarded as seafood consumption of women of childbearing age in this study, which is regarded as an approximation of the seafood consumption of pregnant Japanese women because statistics of pregnant women are limited in the NHNS data.

### 2.4. Effect of Seafood Consumption on Infant IQ

An increase and decrease in IQ of infant is expected due to maternal DHA and MeHg intake during pregnancy, respectively, and net IQ change was estimated by the following Equation (1):

(1) $$\left[net\;IQ\;change\right]=\left[DHA\times 0.04\right]+\frac{\left[MeHg\times 9.3\times \left(-0.18\;or-0.7\right)\right]}{53.2}\cdots \cdots$$

where *DHA* is the daily intake of DHA (mg/day); 0.04 is the slope of regression between DHA intake and IQ; *MeHg* is the daily intake of MeHg (μg Hg/day); 9.3 is the factor converting from daily intake to maternal hair Hg level; −0.18 or −0.7 are the center value and upper bound estimate of regression slope between hair Hg level and IQ; and 53.2 is the average body weight of Japanese women of age 20–39.

Equation (1) is a slightly modified version of the equation proposed in an FAO/WHO document [7] in which the daily intake of DHA and MeHg were given as (number of servings of fish per week) × (serving size of 100 g)/7. In addition, in the FAO/WHO equation, DHA intake was calculated from DHA+EPA content multiplied by 0.67 based on the assumption that fish contains DHA:EPA = 2:1 [7], but daily consumption of DHA only was used in Equation (1) of the present study.

## 3. Results

Table 1 shows the averages of the estimated contents of DHA and MeHg of the 13 categories of seafood consumed in Japan. The category was based on that used in the NHNS and the average contents of each category were obtained by averaging the reported contents of DHA and MeHg in individual seafood items. Correction of the difference of MeHg contents in raw and cooked seafood based on the difference in moisture contents was applied when necessary.

**Figure 1:**
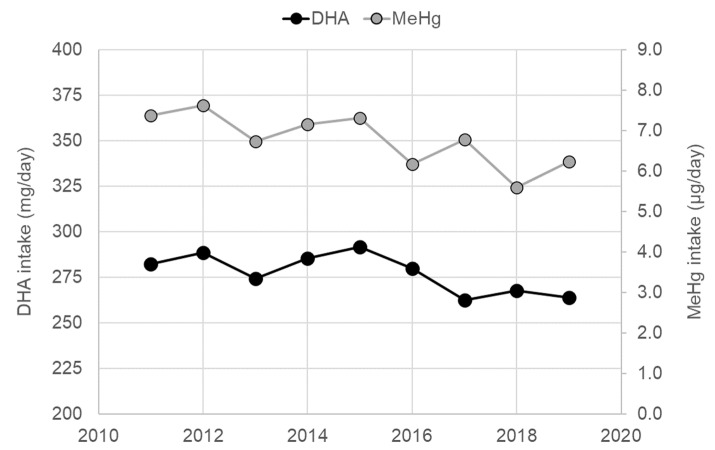
Temporal trend of estimated daily intake of DHA and methylmercury from seafood of Japanese women of childbearing age (20–39 years old).

The intake of DHA and MeHg of Japanese women of childbearing age was calculated by multiplying the average contents in Table 1 by daily consumption (g/day) of each of the 13 categories reported in the NHNS survey of 2011–2019 (see Table S1 for example), and they are plotted in Figure 1. Note that the NHNS survey data on the consumption of each of the 13 seafood categories are available only for this period. Total seafood consumption (sum of the consumption of the 13 categories) of women of childbearing age was available for the period 1999–2019 (Figure S1), and the range was 44 to 73.5 g/day with the mean ± standard deviation being 57.7 ± 9.2 g/day, and the consumption was significantly decreasing during the period 1999–2019 (r = −0.974, *p* < 0.001). Seafood consumption of this age grade (20–39 years old) was approximately 75% of that of general Japanese (men and women of all ages > 1-year-old).

The mean intake of DHA during the 2011–2019 period was calculated to be 277 ± 11 (min–max: 262–292) mg/day and that of MeHg was 6.77 ± 0.77 (5.60–7.62) μg Hg/day. Both of the intakes are linearly decreasing toward the present (Figure 1) in proportion to the decreasing seafood consumption (Figure S1). Coefficients of the negative correlations between year and DHA intake and that between year and MeHg intake were −0.701 and –0.788, respectively (*p* < 0.05). The slope of the regression indicated that intake of DHA and MeHg decreased by 2.8 mg/day and 0.19 μg/day per year, respectively, in the 2011–2019 period.

**Figure 2:**
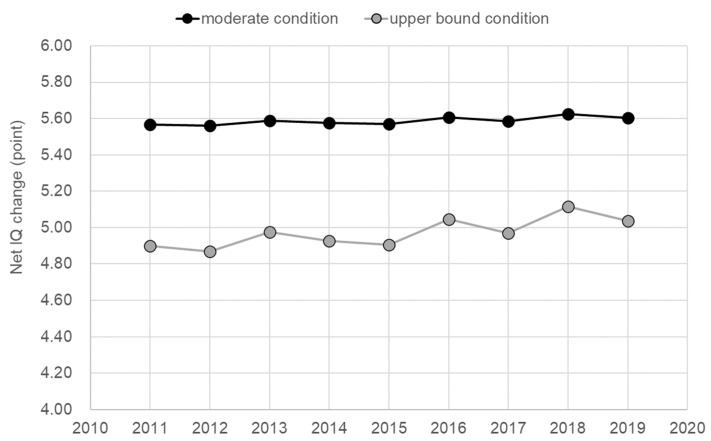
Temporal trend of net IQ change of infant estimated from maternal DHA and methylmercury intake during pregnancy.

Based on the estimated maternal daily intakes of DHA and MeHg, the net IQ change of offspring for each year was calculated by Equation (1). The net IQ change denotes gain/loss of IQ from the infant IQ attained when no seafood was consumed by the mother. In Figure 2, two series of plots are shown: one was calculated by using the center value of the regression slope between hair Hg and IQ (−0.18) (designated “moderate condition” hereafter), and the other was by using upper bound estimate (−0.7) (designated “upper bound condition”) in Equation (1). The calculated net IQ change during 2011–2019 was +5.6 ± 0.0_2_ under the moderate condition and +5.0 ± 0.1 under the upper-bound condition. The net IQ was constantly on the positive side for both conditions, which meant that the seafood intake of the Japanese women of childbearing age resulted in the IQ gain of offspring, and the between-year variation of the net IQ change was small. Under the upper bound condition, net IQ change, i.e., IQ gain, seems to increase as seafood consumption decreases. This trend was not apparent by visual inspection of Figure 2 when the moderate condition is assumed.

## 4. Discussion

It is generally believed that seafood is a healthy food. Therefore, the recent steadily decreasing trend of seafood consumption in Japan (Figure S1) has raised concern about unfavorable health consequences. However, it is sometimes overlooked that seafood contains toxic substances at higher levels, so consumption of seafood may be unfavorable for health. From this particular viewpoint, the recent decrease in seafood consumption may be beneficial for health. The present study looked at DHA and MeHg as beneficial and unfavorable ingredients of seafood, respectively, and quantitatively evaluated the net effect of decreasing seafood consumption of Japanese women on infant IQ.

As shown in Figure S1, a steady decrease in seafood consumption by the general Japanese population started in the mid-1990s. This steady decrease was also the case with women of childbearing age. The IQ gain of infants born to women of childbearing age did not apparently vary when the moderate condition is assumed (regression slope −0.18 in Equation (1)), or even an increasing IQ gain was observed when the upper bound condition was assumed (regression slope −0.7 in Equation (1)) (Figure 2): this means that the decreasing trend in seafood consumption of Japanese women of childbearing age can have even a beneficial effect for infant IQ gain. The latest IQ gain estimate is more than the estimate of 2011 by 0.02 or 0.14 points. If we assume that seafood consumption of women of childbearing age is 75% of that of general Japanese (mean of 1999–2019), the net IQ change of infants at the maximum seafood consumption in 1997 (98.2 g/day) is estimated to be 5.48 and 4.55 points for moderate and upper bound conditions, respectively: the estimated IQ gain at 2019 (5.60 and 5.04) is greater by 0.12 and 0.49 points for the moderate and upper bound condition, respectively, than that at the greatest seafood consumption. This trend of increasing IQ gain while decreasing seafood consumption is due to the fact that seafood consumption by Japanese women is still sufficiently abundant to give beneficial effects on infant IQ. The FAO/WHO assumed that regression between DHA intake and IQ was not linear across the full DHA intake range, but saturation takes place: 5.8 points was the estimated upper limit of IQ gain, which is based on Oken et al. [16]. This means that IQ increase takes place until DHA intake reaches 145 mg/day, when the regression slope 0.04 in Equation (1) is considered, but no further increase is expected when the intake exceeds this level. The estimated DHA intake from seafood was around 280 mg/day for Japanese women of childbearing age during the last 10 years. Therefore, a decrease of seafood consumption only had a decreasing adverse effect on infant IQ from maternal MeHg intake, in other words, a decrease of seafood consumption of the Japanese women of childbearing age so far has had a beneficial effect on infant IQ.

As mentioned, the estimated IQ gain of infants in 2019 was greater than that in 1997, when the decrease in seafood consumption started, by 0.49 points at the maximum estimation. It is well recognized that IQ in childhood is positively associated with economic productivity in adulthood. Grosse and Zhou [17] estimated a 1.4% difference in productivity for 1 point IQ difference in the US. If this is applicable to Japan, then a 0.49 point greater IQ gain would have increased the economic productivity of the Japanese by 0.7% in the last two decades. Thus, decreasing seafood consumption in the last two decades might have an economic effect as well.

**Figure 3:**
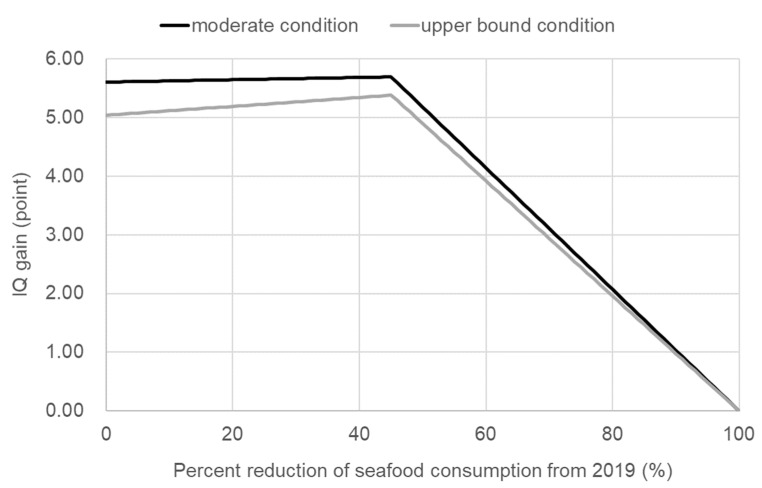
Change of IQ gain of infants according to the reduction of maternal seafood consumption.

It is supposed that the decreasing trend of seafood consumption by the Japanese will continue in the future. Benefits from seafood consumption would eventually disappear when seafood consumption further decreases. Therefore, it is of interest to predict how much reduction in seafood consumption would result in an apparent unfavorable IQ effect. Figure 3 shows the net IQ change of infants as a function of the reduction of maternal seafood consumption. The horizontal axis denotes the percent reduction in the consumption of seafood by women of childbearing age in 2019 (44.0 g/day), and the vertical axis denotes the estimated IQ gain of infants. This simulation indicates that a reduction of seafood consumption of up to 45% of 2019 levels (24.2 g/day) would continue to gradually increase infant IQ gain, and after the reduction exceeds 45%, IQ gain starts to decline with a steep slope, and the IQ gain we have at 2019 would be lost after a 50% reduction of seafood consumption. The steep slope of loss of IQ gain with more than 45% consumption reduction was due to fall of DHA intake below 145 mg/day, the point at which IQ gain starts to decline linearly from 5.8 point. It is predicted that infant IQ gain Japanese infants have at present would be losing when maternal seafood consumption becomes less than 22 g/day. Note that this simulation assumes seafood composition is constant at the composition of 2019 (see Table S1). The simulation result would be different when the composition of seafood changes from that of 2019: for instance, if the consumption of seafood category with higher DHA and lower MeHg content, e.g., “Horse mackerel and Sardine” (Table 1), specifically decreases, the steep decline of infant IQ gain in Figure 3 would start at the lower consumption reduction percentage. It is also noted that this simulation did not take into consideration DHA intake from other source(s) than seafood. The use of DHA supplements would change the simulation result significantly because it provides a significant quantity of DHA, which would relieve the potential effect of decreasing DHA intake associated with decreasing seafood consumption. If a woman uses a DHA supplement, then the steep decline of infant IQ gain would start at a greater consumption reduction percentage. Anyway, this result indicates that there is still a relatively large margin in seafood consumption until the unfavorable effect on infant IQ gain becomes apparent. Note that this estimation is based on infant IQ gain alone, and we need to investigate if the effect of decreasing seafood consumption in Japan has an unfavorable effect on other health outcomes, e.g., cardiovascular disease.

In this study, we assumed that the seafood consumption of pregnant Japanese women is not different from that of women of age 20–39. However, it is possible that a woman may reduce seafood consumption after she becomes pregnant, as the MHLW announced in 2003 that women who are pregnant or who are expected to be pregnant should restrict the frequency of the consumption of specific species of seafood (e.g., certain species of dolphin and whale, tuna, red snapper, etc.) because maternal consumption of these species with elevated MeHg content could bring about adverse effects on infant development [18]. This announcement might have been misunderstood to restrict the consumption of seafood in general. In the United States, a reduction in fish consumption by pregnant women took place after a similar advisory was announced in 2001 by the US EPA: 7.7 servings of fish and shellfish per week before the advisory to 7.1 after the advisory [19]. There is only limited data in Japan on the consumption of seafood by pregnant women that can be compared with non-pregnant women. The NHNS reported food consumption of pregnant and nursing Japanese women in 2011 and 2012. Seafood consumption of pregnant women was 61.7 and 37.7 g/day in 2011 and 2012, respectively, and that of nursing women was 54.1 and 58.6 g/day. These values are to be compared with that of women of childbearing age, i.e., 51.8 and 52.2 g/day, as used for the calculations in the present study. There does not seem to be a significant difference between the seafood consumption of pregnant women and that of women of childbearing age, taking the relatively small sample size of the NHNS data on pregnant women (*n* = 32 and 135 in 2011 and 2012, respectively) into consideration, suggesting that reduced seafood consumption of women after diagnosis of pregnancy was not apparent in Japan. This suggestion justifies the use of seafood consumption data of women of childbearing age for that of pregnant women in this study. In fact, the reported daily DHA intake of Japanese pregnant women was consistent with the estimated intakes of the present study: Shiraishi et al. [20] reported the mean intake of DHA by pregnant Japanese women (n = 262) recruited during 2010–2011 to be 313 ± 181 mg/day based on a diet history questionnaire survey, while the present estimate of women of childbearing age was 283 mg/day in 2011. This may support our notion that the estimated consumption of seafood, or subsequently estimated DHA and MeHg intakes, of women of childbearing age represents that of pregnant Japanese women. A reliable seafood consumption survey of pregnant/non-pregnant women is warranted for the evaluation of diet change after pregnancy and its health consequence for pregnant women themselves and their offspring.

## 5. Conclusions

The decreasing seafood consumption in Japan was not found to have a negative effect on infant IQ gain, but even had a positive effect (more IQ gain). This was because, even though the seafood consumption is decreasing, the consumption is still abundant enough to supply adequate DHA to keep upper-limit IQ gain (+5.8 points), and the decreasing consumption resulted in decreasing adverse effects from MeHg intake. The benefit we have at present can be maintained until seafood consumption is further decreased by 45% of the present level on the assumption that the present seafood composition is constant. It is warranted to examine if the decreasing seafood consumption in Japan has adverse effects on other outcomes, e.g., cardiovascular and cerebrovascular disease/mortality, to further evaluate health effects of the dietary habit change.

## Figures and Tables

**Table 1:** Average contents of DHA and MeHg of 13 categories of seafood.

Category	DHA (mg/g)	MeHg (µg Hg/g)
Horse mackerel and sardine	13.53	0.11
Salmon and trout	9.12	0.06
Sea bream and flatfish	4.06	0.23
Tuna and swordfish	8.33	0.59
Other fish	7.62	0.27
Shellfish	1.78	0.15
Squid and octopus	1.07	0.07
Shrimp and crab	0.59	0.15
Salted fish	5.32	0.07
Canned fish	5.74	0.09
*Tsukudani*	4.40	0.03
Fish paste	0.69	0.05
Fish ham and sausage	4.04	0.06

## Data Availability

The data presented in this study are available on request from the corresponding author.

## Supplementary Materials

The following supporting information can be downloaded at: https://www.mdpi.com/article/10.3390/foods12081674/s1, Figure S1: Temporal trend of daily seafood consumption of the Japanese; Table S1: National average consumption of 13 seafood categories of the Japanese women of age 20–29 and 30–39 (2019).
