# Supplementary material for: Risk and Benefit of Decreasing Seafood Consumption in Japan—Docosahexaenoic Acid, Methylmercury and Infant IQ
Source: Foods. 2023 Apr 17;12(8):1674. doi: 10.3390/foods12081674 (PMC10137566; doi:10.3390/foods12081674)
Supplement: Supplementary file 1 [file foods-12-01674-s001.zip › foods-2337375-supplementary.pdf]

Supplementary materials of “Risk and Benefit of Decreasing Seafood Consumption in Japan—Docosahexaenoic Acid, Methylmercury and Infant IQ” by Shingo Fujimura and Jun Yoshinaga

Figure S1. Temporal trend of daily seafood consumption of the Japanese

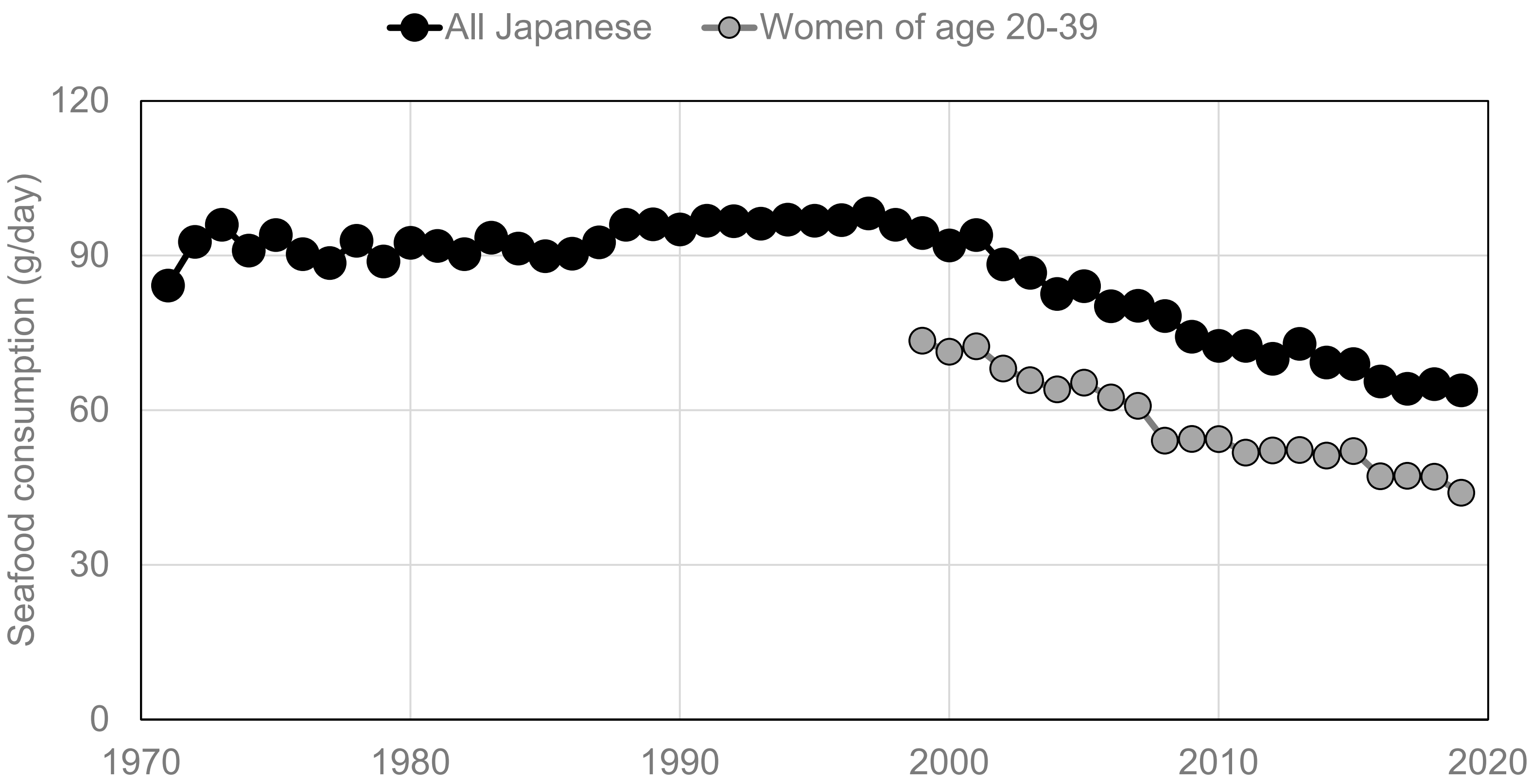

Table S1 National average consumption of 13 seafood categories of the Japanese women of age 20-29 and 30-39 (2019)

Category	Average daily consumption of (g/day)	
	Age 20-29	Age 30-39
Horse mackerel & Sardine	5.4	6.9
Salmon & Trout	4.0	4.4
Sea bream & Flatfish	3.9	2.6
Tuna & Swordfish	2.8	2.5
Other fish	3.0	5.5
Shellfish	0.7	2.8
Squid & Octopus	2.4	1.7
Shrimp & Crab	1.5	3.9
Salted fish	9.9	9.5
Canned fish	2.1	1.8
Tsukudani	0.0	0.2
Fish paste	5.6	4.2
Fish ham & sausage	0.2	0.4
Total	41.6	46.3
